# END-OF LIFE CARE PLANNING: THE ROLE OF FAMILY FOR VIETNAMESE AMERICANS AND FILIPINO AMERICANS

**DOI:** 10.1093/geroni/igad104.3300

**Published:** 2023-12-21

**Authors:** Carson De Fries, My Ngoc To, Fei Sun, Bei Wu, Sue Levkoff, Kaipeng Wang

**Affiliations:** University of Denver, Denver, Colorado, United States; University of Denver, Denver, Colorado, United States; Michigan State University, East Lansing, Michigan, United States; New York University, New York City, New York, United States; University of South Carolina, Columbia, South Carolina, United States; University of Denver, Denver, Colorado, United States

## Abstract

Research has demonstrated a lack of understanding around the significant disparities in end-of-life (EOL) care decisions and culturally sensitive EOL care practices among older Southeast Asian Americans (SAA). Little is known regarding the factors related to the experiences and attitudes toward family involvement in EOL care, especially among these populations. This study explored how family relationships shape attitudes towards and experiences of family involvement in EOL care among older Vietnamese American and Filipino Americans. Three focus groups consisting of one Filipino group (in English), and two Vietnamese groups (one in English, one in Vietnamese) were held with 12 participants aged 55 or above. Interview questions centered around EOL care preferences and experiences and the role of family relationships in EOL care. Participants reported they could trust their children the most with their EOL care wishes, and expressed the preferred place for EOL care to be “home” rather than institutional settings. While some reflected that previous experiences with older family members (e.g., parents, aunts and uncles) facilitated EOL care discussions, others acknowledged resistance from some in participating in these conversations. Cultural and language differences led to participants’ resistance to formalized care and hindrances to EOL care planning. Finally, financial cost, concern about burdening younger generations who “have their own lives”, and difficulty navigating information were cited as barriers to EOL care. Findings from this study can provide improved understanding of the role that family plays in individuals’ EOL care experiences and improve culturally responsive EOL care practices for SAA populations.

